# Neurodevelopmental Syndrome with Intellectual Disability, Speech Impairment, and Quadrupedia Is Associated with Glutamate Receptor Delta 2 Gene Defect

**DOI:** 10.3390/cells11030400

**Published:** 2022-01-25

**Authors:** Anastasia P. Grigorenko, Maria S. Protasova, Alexandra A. Lisenkova, Denis A. Reshetov, Tatiana V. Andreeva, Gilberto De Lima Garcias, Maria Da Graça Martino Roth, Andreas Papassotiropoulos, Evgeny I. Rogaev

**Affiliations:** 1 Center for Genetics and Life Science, Sirius University of Science and Technology, 354340 Sochi, Russia; anast1998@mail.ru (A.P.G.); an_tati@mail.ru (T.V.A.); 2 Laboratory of Evolutionary Genomics, Department of Genomics and Human Genetics, Vavilov Institute of General Genetics, Russian Academy of Sciences, 119333 Moscow, Russia; maria2100@list.ru (M.S.P.); summoner-yuna@yandex.ru (A.A.L.); reshetovdenis@gmail.com (D.A.R.); 3 Department of Psychiatry, UMass Chan Medical School, Shrewsbury, MA 01545, USA; 4 Center for Genetics and Genetic Technologies, Department of Genetics, Faculty of Biology, Lomonosov Moscow State University, 119192 Moscow, Russia; 5 Catholic University of Pelotas, Pelotas 96015-560, RS, Brazil; gilberto.garcias@gmail.com (G.D.L.G.); gmartinoroth@yahoo.com.br (M.D.G.M.R.); 6 Federal University of Pelotas, Pelotas 96010-610, RS, Brazil; 7 Transfaculty Research Platform, University of Basel, CH-4055 Basel, Switzerland; andreas.papas@unibas.ch; 8 Psychiatric University Clinics, University of Basel, CH-4055 Basel, Switzerland

**Keywords:** disequilibrium syndrome, quadrupedal locomotion, speech, congenital cerebellar ataxia, mental retardation, intellectual disability, cerebellum hypoplasia

## Abstract

Bipedalism, speech, and intellect are the most prominent traits that emerged in the evolution of *Homo sapiens*. Here, we describe a novel genetic cause of an “involution” phenotype in four patients, who are characterized by quadrupedal locomotion, intellectual impairment, the absence of speech, small stature, and hirsutism, observed in a consanguineous Brazilian family. Using whole-genome sequencing analysis and homozygous genetic mapping, we identified genes bearing homozygous genetic variants and found a homozygous 36.2 kb deletion in the gene of glutamate receptor delta 2 (*GRID2*) in the patients, resulting in the lack of a coding region from the fifth to the seventh exons. The *GRID2* gene is highly expressed in the cerebellum cortex from prenatal development to adulthood, specifically in Purkinje neurons. Deletion in this gene leads to the loss of the alpha chain in the extracellular amino-terminal protein domain (ATD), essential in protein folding and transport from the endoplasmic reticulum (ER) to the cell surface. Then, we studied the evolutionary trajectories of the *GRID2* gene. There was no sign of strong selection of the highly conservative *GRID2* gene in ancient hominids (Neanderthals and Denisovans) or modern humans; however, according to in silico tests using the Mfold tool, the *GRID*2 gene possibly gained human-specific mutations that increased the stability of *GRID2* mRNA.

## 1. Introduction

A very rare case of cerebellar hypoplasia, quadrupedia or cerebellar ataxia, mental retardation, and disequilibrium syndrome (CAMRQ) or disequilibrium syndromes (DSs) is characterized by the lack of capacity for bipedal locomotion, ataxia, intellectual disability, and speech disturbance. A similar phenotype was described in several consanguineous families in Turkey [1,2,3,4] and Iraq [5]. Despite similar clinical manifestations in all of the unrelated families described in the literature, this form of neurodevelopmental disorder is heterogeneous and is caused by mutations in different genes. So far, six genes with missense mutations which lead to a lack of bipedal locomotion have been previously described: *VLDLR* (low-density lipoprotein receptor) and *RELN* (reelin) genes, both involved in neuronal migration in the developing brain [2,6]; the *CA8* (carbonic anhydrase VIII) gene, which participates in the modulation of intracellular calcium signaling in the cerebellum [5]; the *WDR81* (WD repeat domain 81) gene, which is highly expressed in Purkinje and photoreceptor cells and is involved in endolysosomal trafficking [7]; the *ATP8A2* (aminophospholipid transporter-like ATPase) gene, which is responsible for the transport of aminophospholipids via the lipid flipping process [8]; and the *TUBB2B* (tubulin beta 2B class IIb) gene, which encodes a beta isoform of tubulin—an essential component of the microtubule cytoskeleton [9].

In this study, we performed a comprehensive genetic analysis of a neurological case of quadrupedal locomotion with intellectual disability and the complete absence of speech, found in a Brazilian family [10]. We described the genetic cause of the disease in this family and analyzed the contribution of the *GRID2* gene to bipedalism and speech development.

## 2. Materials and Methods

**Figure 1:**
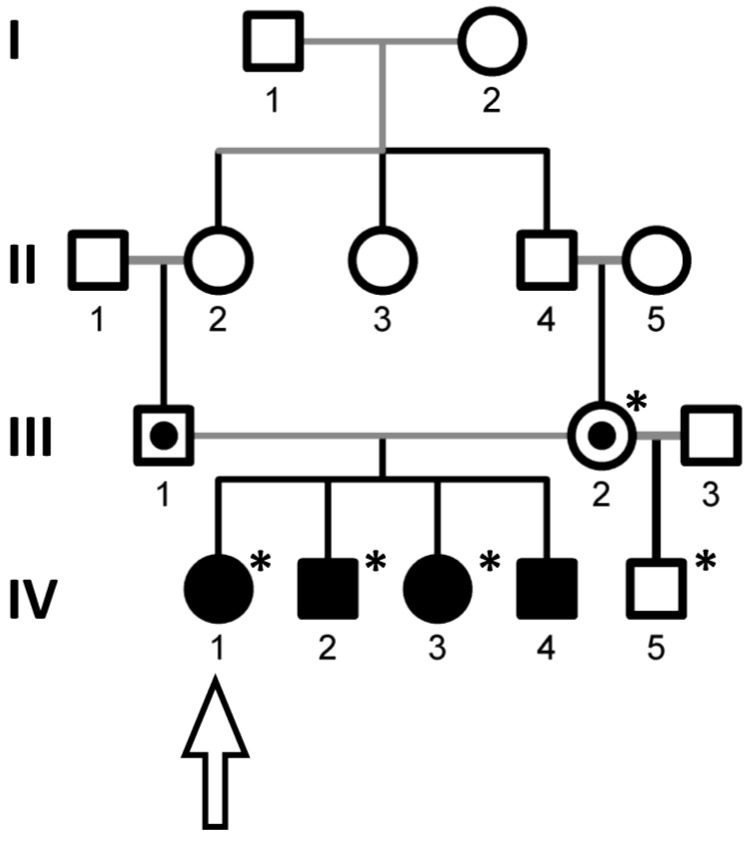
Four generations (I - IV) of Brazilian family are shown. Solid symbols indicate the affected individuals; dotted circles—married cousins, heterozygous obligate carriers; open symbols—unaffected individuals; asterisks—individuals for whom genetic analysis was performed; arrow—patient for whom whole-genome sequencing was obtained.

This family case was previously described [10]. Briefly, due to a cousin marriage, four individuals with neurological pathology were born (Figure 1). All patients (two women and two men) exhibited an absence of the ability to walk without support after a particular development milestone (quadrupedal locomotion), the inability to stand up without support, divergent strabismus, and seizures. External characteristic features include hirsutism, coarse facial features, short and small hands and feet, a small stature, and normal secondary sex characteristics. All patients had intellectual disability and were unable to speak. However, they exhibited friendly behavior towards other people and exhibited no signs of autism. The patients have a healthy stepbrother from the second non-consanguineous marriage of their mother.

The study protocol was approved by the Ethics Committee of the Vavilov Institute of General Genetics, the Russian Academy of Sciences, in accordance with the regulations and guidelines outlined in the Declaration of Helsinki. Blood samples of the members of the Brazilian family were collected with their informed consent.

DNA was extracted using a QIAamp DNA Blood Midi Kit (Qiagen, Hilden, Germany). SNP profiling was performed using the Affymetrix Genome-Wide Human SNP Array 6.0 (Affymetrix, Santa Clara, CA, USA). Homozygosity analysis was performed using Homozygosity Mapper [11]. We obtained the data from Genome-Wide Human SNP Array 6.0 for three affected children (IV-1, IV-2, and IV-3), the unaffected mother (III-2), and the unaffected stepbrother (IV-5). We used the following parameters to analyze the genotype data: require genetic homogeneity—true; allele frequencies—HapMap CEPH (European origin); limit block length—empty; only count blocks longer than 0. The largest max homozygosity score was achieved for three of the affected patients (IV-1, IV-2, and IV-3). A DNA library was prepared using an Illumina Paired-End DNA Sample Prep Kit (Illumina, San Diego, CA, USA). High-throughput sequencing was performed on the Illumina HiSeq2000 platform. Raw reads were aligned to human reference genome GRCh37 using a BWA tool [12,13] with 90× coverage. Variants were called using GATK [14,15,16] and Pindel [17] and were annotated using a Variant Effect Predictor [18]. All rare polymorphisms and mutations were selected using data for 1000 genome sequences [19] and linked to the homozygosity regions. Candidate variants were validated via Sanger sequencing performed on an Applied Biosystems 3730xl DNA Analyzer (Thermo Fisher Scientific, Waltham, MA USA). Oligonucleotide sequences used for validation analysis are listed in Table S3. For the validation of *GRID2* gene 3 oligonucleotides, one direct sequence, and two reverse sequences, one corresponding to the deleted region (345 bp wild type fragment) and another to the flank region (432 bp mutant fragment), were designed (Table S3). Both products were detected in a single PCR reaction.

The molecular evolution of the protein-coding sequence of the *GRID2* gene was studied using a primate dataset for 23 representatives, and a Rodentia (1 representative) species as an outgroup. Coding sequences of the studied genes were obtained from the NCBI Reference Sequence Database [20], Ensembl [21], and the UCSC Genome Browser [22]. Only species with good-quality protein-coding sequences were analyzed. The selected sequences were translated and aligned using MUSCLE, as implemented in MEGA v6 [23]. A phylogenetic tree was reconstructed according to a previous study carried out on primates [24]. Evolutionary analysis was performed using the CodeML program in the PAML package v4.8 [25,26]. Posterior probabilities were estimated using a Bayes Empirical Bayes (BEB) test implemented in CodeML. The FMutSel-F model of codon substitution [27] was chosen as the best-fitting codon model for the analysis.

The influence of the synonymous variants on mRNA thermodynamic stability was analyzed via the estimation of alterations in Gibbs free energy using Mfold software with default parameters [28,29,30]. Gibbs free energy was calculated for the fragments of 25, 51, 75, 151, and 301 nucleotides (nt) and full-length coding sequences (CDS). Only molecules with a minimal value of Gibbs free energy were analyzed. The level of significance was estimated using the Sign Test.

## 3. Results

### 3.1. Genetic Study

**Figure 2:**
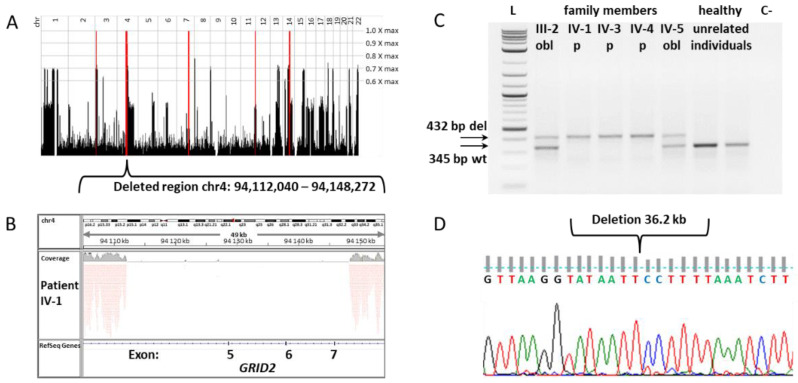
Identification of deletion in *GRID2* gene in consanguineous Brazilian family: (**A**) Loci of homozygosity in three patients (marked in red) (IV-1, IV-2, and IV-3); (**B**) visualization of genomic region of deletion covering 5–7 exons of *GRID2* gene in whole-genome sequencing data of the patient IV-1 (Integrative Genomics Viewer image); (**C**) electropherogram of PCR validation of the deletion in *GRID2* gene: L—DNA ladder. Due to the large deletion region of 36.2 kb, the PCR reaction was performed using two primer pairs with one common forward and two different reverse oligonucleotides. The first primer pair annealed on flanking genomic sequences across deletion and produced a 432 bp fragment corresponding to patients bearing 36.2 kb deletion. The second primer pair annealed on the 5′flanking region and on a genomic sequence within the deletion region, and produced a 345 bp fragment found in healthy individuals. Both PCR products were identified in obligate carriers: mother—III-2, and healthy son—IV-5; (**D**) validation of deletion in *GRID2* gene via Sanger sequencing.

To determine genetic loci linked to the pathogenic mutation in the Brazilian consanguineous family (Figure 1), we performed homozygosity mapping in three affected individuals (IV-1, IV-2, and IV-3), their healthy mother (III-2), and healthy stepbrother (IV-5) using the Affymetrix Genome-Wide Human SNP Array 6.0. Homozygosity Mapper [11] identified five homozygous regions with a maximum homozygosity score of 3000 for the affected patients (IV-1, IV-2, and IV-3) (Figure 2A, Table S1). Next, whole-genome sequencing analysis was performed for one patient (IV-1). A total of 3.1 billion reads (95% of all reads generated) were mapped to the human reference genome, GRCh37, with a 90-fold coverage depth [12,13]. In total, 5.2 million variants were called using the GATK and Pindel tools [14,15,16,17]. We also identified genes with a homozygous variant in the region with a low homozygous score (Table S2C).

In this patient, all rare variants with a global Minor Allele Frequency [19] lower than 5% were selected; 3780 out of the list were located in the coding region of the genes. However, homozygosity regions do not include genes that were previously identified in quadrupedal disorder (*VLDLR*, *WDR81*, *CA8*, *ATP8A2*, *RELN*, and *TUBB2B*). We checked all homozygous rare variants in these genes. We only found one homozygous variant, rs140526335 (MAF 0.0005), in the *VLDLR* gene. CADD prediction placed this variant among the 1% most deleterious substitutions in the human genome. However, using SIFT and PolyPhen programs, the variant was predicted as “tolerated” (0.142) and “benign” (0.083), respectively (Table S2A). Most importantly, the inheritance of this variant in the family’s relatives did not correspond to the disease status (heterozygous variant in patient IV-2). Therefore, this variant was excluded from further analysis.

Next, we analyzed the variants located in regions with high homozygosity based on population frequency, gene expression in the developing brain and cerebellum, and affecting protein structure (Table S1). In the largest region with maximum homozygosity, variants in the *COQ2*, CDS*1*, and *GRID2* genes were selected (Table S2B). All variants were verified via PCR and Sanger sequencing. The homozygous state of the variants was confirmed in all of the examined patients from the Brazilian family, while their mother and stepbrother were confirmed to be heterozygous. The rs112033303 variant, which was predicted to be among the 1% most deleterious substitutions from the human genome via CADD prediction, was found in the *COQ2* gene. The *COQ2* gene codes a coenzyme, Q2 4-hydroxybenzoate polyprenyl transferase, and is widely expressed in different tissues [31]. Mutations in the *COQ2* gene lead to multiple system atrophy [32,33,34,35] and cerebellar ataxia [36]. The rs112033303 variant in the *COQ2* gene leads to a stop codon in the 22nd amino acid of a signal peptide with the longest protein isoform. However, several transcripts that encode full-length protein isoforms with alternative start codons are located downstream of this variant (Table S2B). The minor variant, rs112033303, is present in the population and is most common in Iberian individuals, with an MAF of 0.056119. Several studies in the ClinVar database provide evidence of null impact or a likely benign status for this variant [35,37,38]. The wild-type variant of rs112033303 that is distributed between placental mammals, particularly in primates, is not highly conserved, suggesting that minor allele is probably not deleterious.

Another nucleotide substitution in the *CDS1* gene, G > C (rs118099717), causes glycine to change to alanine at an evolutionarily conserved position, the wild type variant is present in most placental animals and reptiles—except lizards, in which alanine is present. A minor variant is present in the human population with an MAF of 0.001, but no carrier with a homozygous state exists [19,39]. For this variant, its functional effect was predicted as “tolerated” (0.844) via SIFT and as “benign” (0.002) via PolyPhen; moreover, CADD predicted that it was among the 10% most deleterious substitutions in the human genome. This gene is widely expressed in different adult and fetal tissues, particularly in the colon, epididymis, duodenum, placenta, small intestine, thyroid, and skin [31,40], and is highly expressed in the cerebellum at the 16th week post conception [41]. Although no gross impairments in other tissues were reported in the Brazilian patients, some modifying effect of the CDS1 gene on the overall phenotype cannot be excluded.

**Figure 3:**
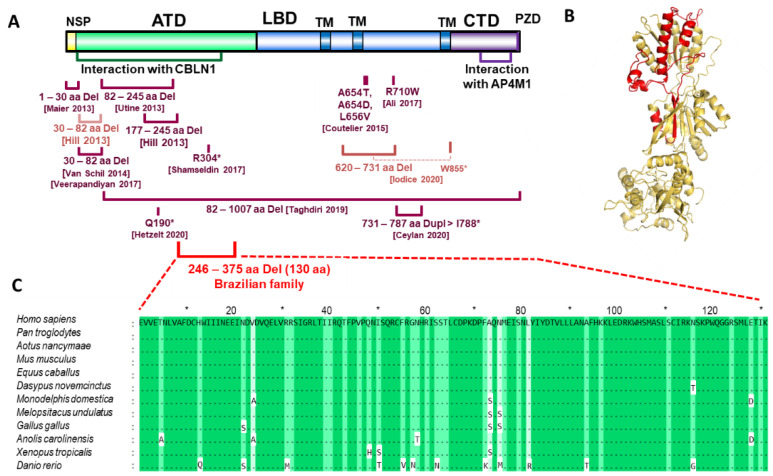
Representation of deleted region in glutamate receptor delta 2 (*GRID2*): (**A**) The glutamate receptor delta 2 precursor consists of N-terminal signal peptide (NSP), extracellular amino-terminal (ATD), ligand binding domain (LBD), 3 transmembrane domains (TMs), and C-terminal intracellular domain (CTD) with PZD domain [55]; protein structure created using IBS [56]. Pathogenic variants are indicated in reported family cases: in red—deletion of exons 5–7 encoding a part of ATD found in Brazilian patients; in dark red—homozygous variants (Del—deletions, Dupl—duplications, nonsense, and missense) reported previously in other studies; in light red—heterozygous variants (compound variants joined by broken line, reported previously in other studies); *—stop codon; (**B**) the prediction of spatial structure of GRID2 receptor monomer (NP_001501.2) via Phyre2 was visualized using Pymol software. Deleted region contains part of a large extracellular alpha chain domain and one beta strand (marked in red); (**C**) deleted region conservation across animals visualized in GeneDoc program [57]; evolutionary conserved amino acids are indicated in green. Protein CDSs were obtained from the NCBI nucleotide database and aligned using the MEGA6 software [23].

The third potential variant identified in chromosome region 4q22 in the largest homozygous region was the deletion region in the *GRID2* gene (hg19 chr4:g.94,112,040_94,148,272del) (Figure 2B, Table S2B). The deletion region covers exons 5–7 of the longest isoform and affects all other isoforms. In-frame deletion affects 130 amino acids (V246–K375) in the extracellular amino-terminal (ATD), alternatively named the N-terminal extracellular leucine/isoleucine/valine-binding protein (LIVBP-like) domain, which is involved in ligand binding [42,43], protein tertiary structure formation, and transport from the endoplasmic reticulum to the cell surface [44] (Figure 3A,B). This region is highly conserved between animals (Figure 3C). Disruptions in the *GRID2* gene were described in several family cases with cerebellar ataxia [45,46,47,48,49,50,51,52,53] and in a mouse model [54] (Figure 3A). Deletion was confirmed to be homozygous in all of the examined patients from the Brazilian family, and heterozygous in their parent, via PCR and Sanger sequencing (Figure 2C,D).

Rare non-synonymous homozygous variants in genes from different genomic regions were also found in several other genes in the patient V-I of this consanguineous family (listed in Table S2C). They could potentially contribute to some pathogenic features in this syndrome associated with major defects caused by *GRID2* deletion.

### 3.2. Evolutionary Analysis of Protein-Coding Sequences

**Figure 4:**
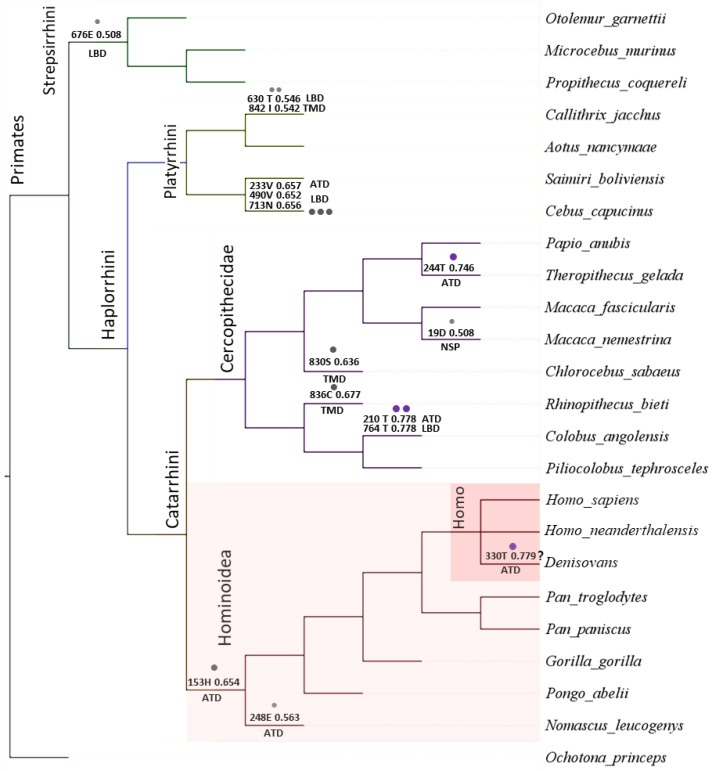
Phylogenetic tree for the primate dataset of *GRID2* gene. Putative sites determined in model A1 using Bayes Empirical Bayes (BEB) method are indicated in branches by dots; amino acid site position with a BEB probability value and protein domain are specified. “?”—The presence of this variant is not unambiguous and could be a result of ancient DNA hydrolytic deamination.

We performed the evolutionary analysis of the *GRID2* gene, associated with quadrupedal locomotion, speech and thinking impairments, in a Brazilian family. The analysis of the protein-coding sequences of the *GRID2* gene among modern humans, ancient hominids, and primates was carried out using the CodeML program, part of the PAML package [25,26]. The GRID2 protein-coding sequence is highly conserved among vertebrates, reaching the highest conservation (93–99%) in primates. The rate of the accumulation of changes in the protein-coding sequence of the *GRID2* gene varied across the primates’ taxa in accordance with the statistically significant alternative hypothesis of the molecular clock test implemented by CodeML (*p* value < 0.0025, 2∆lnL −45.257462, df 22). Thus, we assessed possible changes in selective pressure on the reconstructed phylogenetic tree of the primate dataset by consecutively assigning each crown branch as the foreground branch during CodeML analysis. The ω value across branches was investigated under the one-ratio model (M0), which assumed an equal ω value for all branches, and the free-ratio model (M1), which assumed an independent ω ratio for each branch. Even if the hypothesis of the uniformity of evolutionary rates (molecular clock) was rejected, the M0 and M1 models were not statistically different (Table S4). The ω omega of M0 was estimated to be 0.027, indicating a strong purifying tendency. Then, the primate dataset of the *GRID2* gene was investigated using branch-site-specific model A (MA0/MA1). The MA1 model was rejected, and the MA0 model was the best fit in all of the branches (Table S5). Additionally, no putative sites were indicated under positive selection using the Bayes Empirical Bayes (BEB) method. Model A only indicated sites with low foreground probability (0.5 < BEB < 0.8) in the ATD (six sites) and in the LBD (eight sites) (Figure 4, Table S5). In the ape (Hominoidea) branch, one site with low foreground probability (153 H 0.654) was indicated in ATD; however, this is the only site that distinguishes all monkeys under the category of apes from other primates. The protein-coding sequence of the closest living relatives to humans (*Pan troglodytes* and *Pan paniscus*) differs by 13 substitutions, 12 of which are synonymous (Figure S1). Amino acid sequences of *Pan troglodytes* and *Pan paniscus* differ from *Homo sapiens* by one nonsynonymous substitution; this substitution is localized in the signal peptide and does not affect the main functional domains. No substitutions between *Homo sapiens* and Neanderthals were found. The Denisovan coding sequence for this gene differs from that for *Homo sapiens* by one synonymous substitution and one potentially heterozygous non-synonymous substitution (rs373866971, MAF 0.000039 gnomAD; however, due to the nucleotide change, C to T, and the ratio of reference and minor allele 21/3, this may be a technical mistake recorded in the results regarding hydrolytic damage to ancient DNA) (Figure S1). This non-synonymous substitution has a low level of BEB probability (330 T 0.779) and is localized in the region that is deleted in the Brazilian patients. It is possible that this Denisovan-specific variant negatively affects protein function, as the ancestral variant is highly conservative. This variant has a very low frequency in modern human populations and, moreover, is predicted to be “probably damaging” via PolyPhen and as “deleterious” via SIFT.

The best-fitting FMutSel1 model estimated the selection of the synonymous rate; therefore, we analyzed the mRNA stability of *Homo sapiens*, Denisovan, and *Pan troglodytes* CDS using the Mfold tool. In terms of energy, a full-length *Homo sapiens* and Denisovan CDS is more efficient than *Pan troglodytes* CDS: dG −954.5 and −954.4 kcal/mol as opposed to −947 kcal/mol, respectively. As Mfold tools are more effective for use with short sequence fragments, we analyzed regions across two non-synonymous and thirteen synonymous variants over 25, 51, 75, 151, and 301 nucleotides (nt) of *GRID2* CDS (Table S6) [28,58]. Most variants (10 out of 15) were neutral or did not reach a statistically significant difference in Gibbs free energy. The estimation of Gibbs free energy for other variants demonstrated that *Homo sapiens GRID2* CDS has a significantly lower level of Gibbs free energy for the specific variants G^94436426^ and G^94436547^ and a slightly higher level for the variants A^94159638^, T^94316763^, and G^94376878^ as compared to *Pan troglodytes* and Denisovan. To assess the observed trends among Hominoidea, sites with conservative substitutions were selected in Hominini (two substitutions), Homininae (six substitutions), and Hominoidea (five substitutions) (Table S7). Gibbs free energy levels were compared for the ancestor and mutant alleles between the primate taxa. For the majority of sites (11 out of 14), no statistically significant changes in the Gibbs free energy levels were found. At the beginning of the coding sequence of the *GRID2* gene, two sites were identified, located at a close distance from each other, whose synonymous substitutions in Hominini can lead to a statistically significant decrease in Gibbs free energy levels (Table S8). Moreover, the nonsynonymous substitution of N153H in Hominoidea also leads to a statistically significant decrease in Gibbs free energy levels (Figure 4, Table S8). Thus, it is likely that in the branches of the Hominoidea taxon for the *GRID2* gene, the selection of variants that lead to the formation of a more optimal RNA spatial structure is taking place.

## 4. Discussion

Our study expanded the genetic and evolutionary constituent of quadrupedal locomotion and severe intellectual disability in a Brazilian family. The exceedingly rare phenotype that is presented in this case is caused by deletion in the *GRID2* gene. Previously, the disruption of this gene was linked to progressive autosomal recessive ataxia. In all previously described cases, the common clinical signs, including coordination disorder and global development delay, are results of cerebellar atrophy [45,46,47,48,49,51,52,53,59,60] (Table S9). We cannot completely rule out that insufficient neurorehabilitation in the first years of life might contribute to the quadrupedal locomotion in the Brazilian family. In most cases of patients with *GRID2* defects, bipedal locomotion was possible with or without support, despite the gross motor development delay. Here, we focused on patients from a Brazilian family, first described by Garcias and Roth, who possess distinctive features which were not specified in other cases of *GRID2* gene pathology (SCAR18): quadrupedal locomotion, the absence of speech, severe intellectual disability, seizures, and some appearance features (hirsutism, coarse facial features, and stunting). In some other previously reported cases of patients with *GRID2* mutations, speech disorders in patients were characterized by dysarthria without limits in speaking, or consisting of simple sentences. Potentially, other genetic variants could contribute to the morphological or neuropathological features of the “involution” syndrome in the Brazilian family (Table S2). For example, a rare variant was found in the *CDS1* gene located in the same largest homozygous 4q22 region. The *CDS1* is involved in phosphoinositide biosynthesis and lipid droplet biogenesis, and is expressed in the brain and other tissues [31,40,41,61]. However, this genetic variation in *CDS1* is predicted to be functionally insignificant. We also found rare genetic variants in genes from genomic regions with relatively low homozygous scores (Table S2C). Most interestingly, among them is the deleterious missense mutation in a highly evolutionarily conserved site in the *ARNT2* gene. The *ARNT2* gene has a major expression in different brain regions, and was linked to autosomal recessive Webb–Dattani syndrome in one family [62]. However, none of the mutations in these genes corresponded to the expected autosomal recessive inheritance (Table S2C). 

In the Brazilian patients, coordination disorder was accompanied by seizures, which have not been noted in any other described cases; moreover, there is no mention of hirsutism and stunting in previously described cases regarding *GRID2* gene pathology [45,46,47,48,49,51,52,53,59,60]. We also cannot exclude the possibility that their symptoms of hirsutism are related to other genetic factors. The deletion removes exons 5–7 of the *GRID2* gene which encode half of the ATD adjacent to the LBD. Such deletion affects all of the predicted protein isoforms of GRID2, whereas in the majority of the previously reported cases [45,46,47,48,49,52,53,59,60] except one [51], the observed mutations do not lead to the impairment of all of the predicted isoforms. The deletion observed in the Brazilian patients partially overlaps with the deletion observed in ho-4J mice [54], which also exhibited ataxia, abnormal motor learning, and impaired balance and coordination.

Anthropological analysis attributes bipedalism to Australopithecus [63,64]. Thus, we investigated the evolutionary involvement of the *GRID2* gene in the regulation of locomotion, speech ability, and intellect across primates. The GRID2 protein sequence is not altered in humans as compared to chimpanzees; moreover, there are no branches with statistically significant evidence of positive selection. However, synonymous selection in *Homo sapiens* CDS was predicted using the mutation-selection model. An additional test of mRNA stability predicted several significant alterations in Gibbs free energy levels for *Homo-sapiens*-specific synonymous substitutions in highly conserved positions. Two substitutions in the central part of the *GRID2* CDS lead to an increased level of Gibbs free energy, while another two, localized in the second part of the CDS, decrease the level of Gibbs free energy. Moreover, additional analyses across Hominoidea taxa also showed a tendency toward a decrease in the level of Gibbs free energy. Changes in the spatial structure of RNA affect the further transport and translation of the protein; the formation of the optimal spatial structure of RNA contributes to better production, accuracy, and folding of the synthesized protein [65,66]. Interestingly, *GRID2* achieves a high level of expression in Purkinje cells, starting from embryonic development and remaining at a high level throughout an individual’s life, and is almost absent in other parts of the brain (Figure S2). Additionally, the expression of the *GRID2* gene is conserved among mammals (Figure S3). A low level of expression is observed in other parts of the brain, including the frontal cortex, where *GRID2* is expressed in the cortex neurons of layer five, which is involved in the formation of motor functions (Figure S4). As shown in mouse development, one of the main functions of the GRID2 receptor in Purkinje cells is synaptogenesis. D-Serine-dependent signaling underlies the long-term depression of cerebellar parallel fiber–Purkinje cell (PF–PC) synapses and motor coordination, which are enabled through interaction with pre-synaptic β-neurexin-1 (β-NRX1) via Cbln1 [42,43,67]. An ATD in the GRID2 receptor is essential for interaction with Cbln1 and to form a complex with β-NRX1 [67]. The loss of ATD, as a result of deletion in the Brazilian family, possibly abolished this interaction. Thus, the Brazilian family represent a case of the disruption of bipedal locomotion, speech, and cognitive functions associated with gene defect of Purkinje cells in the cerebellum.

## 5. Conclusions

In this study, we described a very rare clinical syndrome with quadrupedal locomotion, speech, and learning disabilities caused by a mutation in the highly conserved *GRID2* gene, and studied the evolutionary value of this alteration. The results of this study indicate a high degree of evolutionary conservation of the protein-coding sequence of the *GRID2* gene. The disruption of these regions leads to the loss of bipedal locomotion and affects ancient, evolutionary biological functions in the brain that are responsible for locomotion, cognitive function, and speech. Importantly, other genetic events, such as synonymous substitution, which influences mRNA spatial structure and stability, could have an additional or modifying effect on disease manifestation.

## Data Availability

The data presented in this study are available on request from the corresponding author.

## Supplementary Materials

The following are available online at https://www.mdpi.com/article/10.3390/cells11030400/s1. Figure S1: Evolutionary analysis of nucleotide substitutions in CDS of *GRID2* gene in Homo sapiens and close relatives; Figure S2: Expression of *GRID2* gene in different brain regions in Homo sapiens; Figure S3: Expression of *GRID2* gene in the cerebellum in three mammalian species; Figure S4: Single-cell expression of *GRID2* gene in the cerebellum and frontal cortex of adult mice; Table S1: Regions of homozygosity found by Homozygosity Mapper in patients (IV-1, IV-2, IV-3); Table S2: List of candidate genes detected in Brazilian family case; Table S3: Oligonucleotide sequences used to validate candidate variants; Table S4: Evolutionary analysis of primate protein-coding sequences of *GRID2* gene using one-, free-, and two-branch-specific models; Table S5: Evolutionary analysis of primate protein-coding sequences of *GRID2* gene using branch-site-specific model A; Table S6: Gibbs free energy values for mRNA fragments of the GRID2 gene and their change depending on nucleotide variants, calculated via Mfold; Table S7: Gibbs free energy values for mRNA fragments of the *GRID2* gene in different species of primates, calculated via Mfold; Table S8: Gibbs free energy values for mRNA fragments of the *GRID2* gene and their change depending on nucleotide variants in different primate taxa, calculated via Mfold; Table S9: Comparison of the clinical characteristics of patients with different mutations in *GRID2* gene.
