# Supplementary material for: Neurodevelopmental Syndrome with Intellectual Disability, Speech Impairment, and Quadrupedia Is Associated with Glutamate Receptor Delta 2 Gene Defect
Source: Cells. 2022 Jan 25;11(3):400. doi: 10.3390/cells11030400 (PMC8834146; doi:10.3390/cells11030400)
Supplement: Supplementary file 1 [file cells-11-00400-s001.zip › cells-1522047_Supplemental_Data.pdf]

Supplemental Tables

Table S1. Regions of homozygosity found by HomozygosityMapper [1] in patients (IV-1, IV-2, IV-3).

Score	chr	From (bp)	To (bp)	From SNP	To SNP	Length, bp
3000	4	78,505,578	94,129,968	rs355687	rs994011	15,624,390
3000	7	103,397,404	113,529,740	rs694894	rs8180864	10,132,336
3000	14	56,348,506	66,419,099	rs28496257	rs12880549	10,070,593
3000	3	10,627,561	13,516,646	rs12490065	rs2597506	2,889,085
2452	14	66,448,348	69,163,110	rs1147455	rs8013046	2,714,762
3000	11	132,719,646	134,944,770	rs4575282	rs11224232	2,225,124
2687	4	94,142,499	96,324,373	rs1875705	rs12510417	2,181,874

Table S2. List of candidate genes detected in the genome of Brazilian family patient (IV-1).

Gene	Coordinates	ID	Variant**	Genotype	Exon	Amino acid substitution	Minor Allele Frequency in population [2]		SIFT	POLIPHEN	CADD (PHRED score)***
							Global	Max MAF			
**A. Variants in potential genes********											
VLDLR	chr9: 2,639,898	rs140526335	A>G	Homoz.	3	N81S	0.0024	0.0196 ITU	Tolerated (0.142)	Benign (0.083)	26.6
**B. Variants in region of maximum homozygosity**											
COQ2	chr4:84,206,004	rs112033303	T>A	Homoz.	1	R22*	0.01	0.0561 IBS			25.8
CDS1	chr4:85,525,421	rs118099717	G>C	Homoz.	2	G48A	0.0004	0.0097 CHB	Tolerated (0.844)	Benign (0.003)	18.43
GRID2	chr4:94,112,040-94,148,272	-	36.2 kb deletion, in frame	Homoz.	5-7	Deletion of 130 aa (V246-K375)	-	-	-	-	-
**C. Variants in small regions of homozygosity with potential minor effect********											
PUM3	chr9:2,831,011	rs1241779268	T>C	Homoz	7	S210G	0.0002	0.0052 ACB	Tolerated (0.05)	Benign (0.412)	23.5
ARNT2	chr15:80,866,557	rs750637347	C>G	Homoz	13	S462C	0.0002	0.0047 IBS	Deleterious (0.01)	Possibly damaging (0.894)	23.6
ODF3L2	chr19:467,696	rs756671415	C>T	Homoz	3	R101H	0.0004	0.0118 PEL	Deleterious (0)	Possibly damaging (0.98)	26.9

ITU — Indian Telugu from the UK; IBS — Iberian population from Spain; CHB — Han Chinese in Beijing, China; ABS — African Caribbean in Barbados; PEL — Peruvians from Lima, Peru. Homoz. – homozygous state.

*Stop codon

**Variants are reported in the forward orientation.

***The CADD PHRED score more than 10 indicates the 10% most deleterious substitutions from the human genome; more than 20 indicates the 1% most deleterious substitutions from the human genome [3].

******** The inheritance of these variants in the family's relatives did not correspond to the disease status. Genotyping analysis by PCR and Sanger Sequencing demonstrated:

- rs140526335 variant in *VLDLR* gene is in homozygous state of minor allele in two patients (IV-1 and IV-3), in heterozygous state in one patient (IV-2), mother (III-2) and healthy step-brother (IV-5);

- rs1241779268 variant in *PUM3* gene is in homozygous state of minor allele in two patients (IV-1 and IV-2), in heterozygous state in one patient (IV-3), mother (III-2) and healthy step-brother (IV-5);
- rs750637347 variant in *ARNT2* gene is in homozygous state of minor allele in two patients (IV-1 and IV-2), in heterozygous state in mother (III-2) and healthy step-brother (IV-5), in homozygous state of wild allele in one patient (IV-3);
- rs756671415 variant in *ODF3L2* gene is in homozygous state of minor allele in one patient (IV-1), in heterozygous state in one patient (IV-3), mother (III-2) and healthy step-brother (IV-5), in homozygous state of wild allele in one patient (IV-2);

Table S3. Oligonucleotide sequences used to validate candidate variants.

Oligonucleotide name	Sequence (from 5'end)
VLDLR_For	AGTGCCCATTGACTCAGCTT
VLDLR_Rev	TGAACCATCTTCGCAGTCAG
COQ2_For	GACGAGCTCGGATTGACG
COQ2_Rev	GCCTTTTGCCAATAGAAATCC
CDS1_For	GAATGTATTGGCCAGCCTGT
CDS1_Rev	AGGGGTTCTATCTGAGGATGG
GRID2_flanked_region_For	GACCTACGCTATTTCCTAGCTTT
GRID2_deletion_region_Rev	GCAATTATGCCAGATCATTCTC
GRID2_flanked_region_Rev	TGTAGTCCCAGCTACTCAGGA
PUM3_For	TGTTTAGGTTTGTCTTCTCCAGA
PUM3_Rev	ATGCCAACAAATCAGAAGGTG
ARNT2_For	CCTCTGAGGTATGGGAAAAGTC
ARNT2_Rev	TGTAGGTCTGCTCTTTCCATTG
ODF3L2_For	CTGGTGGGAGTGTCTCGG
ODF3L2_Rev	CTCCTCCGTGTCCCTTGG

Table S4. Evolutionary analysis of primate protein-coding sequences of *GRID2* gene using one, free and two branch-specific models [4,5].

Model		NP	lnL	ω^{M0}			2ΔlnL	P-value	
M0		50	-7471.5742	0.02747					
M1		94	-7445.4004				52.35	0.182	
**Model**	**Branch number**	**NP**	**lnL**	**ω^{M1}**	**ω^{M2} (backgr)**	**ω^{M2} (foregr)**	**2ΔlnL**	**P-value**	**FDR-corrected p-value**
M2	45	51	Min: -7471.57; Max: -7466.60	Min: 0.0001; Max: 0.5641	Min: 0.02573; Max: 0.0315	Min: 0.0001; Max: 115.97	Min: -52.35; Max: -42.40	>0.15	>0.23

Table S5. Evolutionary analysis of primate protein-coding sequences of *GRID2* gene using branch-site-specific model A [6].

		MA0		MA1					
#	Branch	NP	lnL	NP	lnL	2∆lnL	P-value	FDR-corrected p-value	BEB
1	*Homo sapiens*	52	-7447.2991	53	-7447.2991	0.000006	0.4990228	0.5	
2	*Homo sapiens neanderthalensis*	52	-7447.2991	53	-7447.2991	0.000004	0.4992021	0.5	
3	*Homo sapiens ssp 'Denisova'*	52	-7445.3979	53	-7445.3979	0.000004	0.4992021	0.5	330 T 0.779
4	*Homo*	52	-7447.2991	53	-7447.2991	0.000002	0.4994358	0.5	
5	*Pan troglodytes*	52	-7447.2991	53	-7447.2991	0.000004	0.4992021	0.5	
6	*Pan paniscus*	52	-7447.2991	53	-7447.2991	0.000004	0.4992021	0.5	
7	*Pan*	52	-7447.2991	53	-7447.2991	0.000004	0.4992021	0.5	
8	*Hominini*	52	-7447.2991	53	-7447.2991	0.000002	0.4994358	0.5	
9	*Gorilla gorilla*	52	-7447.2991	53	-7447.2991	0	0.5000000	0.5	
10	*Homininae*	52	-7447.2991	53	-7447.2991	0.000002	0.4994358	0.5	
11	*Pongo abelii*	52	-7447.2991	53	-7447.2991	0	0.5000000	0.5	
12	*Hominidae*	52	-7447.2991	53	-7447.2991	0.000026	0.4979658	0.5	
13	*Nomascus leucogenys*	52	-7447.2991	53	-7447.0931	0.411976	0.2604841	0.5	248 E 0.563
14	*Hominoidea*	52	-7446.6573	53	-7446.6573	0.000006	0.4990228	0.5	153 H 0.654
15	*Papio anubis*	52	-7447.2991	53	-7447.2991	0	0.5000000	0.5	
16	*Theropithecus gelada*	52	-7445.8937	53	-7445.8937	0	0.5000000	0.5	244 T 0.746
17	*Papio&Theropithecus*	52	-7447.2991	53	-7447.2991	0	0.5000000	0.5	
18	*Macaca fascicularis*	52	-7447.2991	53	-7447.2991	0.000004	0.4992021	0.5	
19	*Macaca nemestrina*	52	-7446.5422	53	-7446.2213	0.641836	0.2115234	0.5	19 D 0.508
20	*Macaca*	52	-7447.2991	53	-7447.2991	0.000002	0.4994358	0.5	
21	*Papionini*	52	-7447.2991	53	-7447.2992	0.000130	0.4954515	0.5	
22	*Chlorocebus sabaeus*	52	-7446.7419	53	-7446.7419	0	0.5000000	0.5	830 S 0.636
23	*Cercopithecinae*	52	-7447.2991	53	-7447.2991	0	0.5000000	0.5	
24	*Rhinopithecus bieti*	52	-7446.5335	53	-7446.5335	0	0.5000000	0.5	836 C 0.677
25	*Colobus angolensis*	52	-7444.0159	53	-7443.8565	0.318818	0.2861594	0.5	210 T 0.778<br>764 T 0.778
26	*Piliocolobus tephrosceles*	52	-7447.2991	53	-7447.2991	0	0.5000000	0.5	
27	*African Colobinae*	52	-7447.2991	53	-7447.2991	0.000084	0.4963437	0.5	
28	*Colobinae*	52	-7447.2991	53	-7447.2991	0.000004	0.4992021	0.5	
29	*Cercopithecidae*	52	-7447.2991	53	-7447.2991	0.000004	0.4992021	0.5	
30	*Catarrhini*	52	-7447.2991	53	-7447.2991	0.000002	0.4994358	0.5	
31	*Callithrix jacchus*	52	-7446.509	53	-7446.5090	0	0.5000000	0.5	630 T 0.546<br>842 I 0.542
32	*Aotus nancymaae*	52	-7447.2991	53	-7447.2991	0	0.5000000	0.5	
33	*Callithrix&Aotus*	52	-7447.2991	53	-7447.2991	0.000006	0.4990228	0.5	
34	*Saimiri boliviensis*	52	-7447.2991	53	-7447.2991	0	0.5000000	0.5	
35	*Cebus capucinus*	52	-7444.042	53	-7444.0420	0.000002	0.4994358	0.5	233 V 0.657<br>490 V 0.652<br>713 N 0.656
36	*Saimiri&Cebus*	52	-7447.2991	53	-7447.2991	0.000002	0.4994358	0.5	
37	*Platyrrhini*	52	-7447.2991	53	-7447.2991	0.000004	0.4992021	0.5	
38	*Simiiformes*	52	-7447.2991	53	-7447.2991	0.000004	0.4992021	0.5	
39	*Microcebus murinus*	52	-7447.2991	53	-7447.2992	0.000168	0.4948293	0.5	

40	*Propithecus coquereli*	52	-7447.2991	53	-7447.2991	0	0.5000000	0.5	
41	*Lemuriformes*	52	-7447.2991	53	-7447.2990	0.000092	0.4961735	0.5	
43	*Otolemur garnettii*	52	-7447.2992	53	-7447.2991	0.000150	0.4951141	0.5	
44	*Strepsirrhini*	52	-7447.2683	53	-7447.2991	0.061648	0.4019550	0.5	676 E 0.508
45	*Primates*	52	-7446.2892	53	-7446.2892	0.000004	0.4992021	0.5	

Table S6. Gibbs free energy values for mRNA fragments of the *GRID2* gene and their change depending on nucleotide variants, calculated by mFold [7].

Chromosome 4 position	93225840	94031933	94031993	94137900	94145790	94159584	94159638	94316763	94344032	94344060	94376878	94411797	94436426	94436547	94690593
rs								rs1385405	rs72668716						
Nucleotide position in CDS	32	564	624	801	989	1188	1242	1251	1458	1488	1611	1866	2058	2178	2592
The species which have ancestral allele	*Hs*	*Pt*	*Pt*	*Hs*	*Hs, Pt*	*Hs*	*Pt*	*Pt*	*Pt*	*Pt*	*Pt*	*Pt*	*Pt*	*Pt*	*Hs*
	dG (kcal/mol)														
**301 nt**															
Homo sapiens	-84.5	-68.0	-75.4	-79.9	-87.9	-89.6	-92.0	-91.0	-79.4	-79.9	-87.6	-76.2	-77.7	-86.3	-84.0
Denisova	-84.5	-68.0	-75.4	-79.9	-87.5	-89.9	-92.3	-91.3	-79.4	-79.9	-87.6	-76.2	-77.7	-86.3	-84.0
Pan troglodytes	-82.9	-67.9	-74.8	-79.5	-87.9	-90.3	-94.3	-93.3	-79.2	-79.1	-87.9	-78.3	-72.0	-80.8	-87.6
**151 nt**															
Homo sapiens	-35.8	-29.6	-29.7	-30.5	-37.4	-32.6	-30.5	-31.3	-37.9	-33.0	-35.5	-36.1	-43.8	-39.0	-39.4
Denisova	-35.8	-29.6	-29.7	-30.5	-36.6	-35.1	-31.5	-32.5	-37.9	-33.0	-35.5	-36.1	-43.8	-39.0	-39.4
Pan troglodytes	-36.0	-28.6	-31.0	-31.7	-37.4	-34.6	-31.8	-33.6	-35.2	-28.9	-36.2	-36.6	-38.7	-38.5	-40.3
**75 nt**															
Homo sapiens	-17.6	-16.6	-11.0	-5.9	-11.0	-11.2	-16.7	-15.0	-11.4	-12.6	-15.5	-17.0	-21.3	-11.7	-18.8
Denisova	-17.6	-16.6	-11.0	-5.9	-11.0	-11.2	-17.0	-15.3	-11.4	-12.6	-15.5	-17.0	-21.3	-11.7	-18.8
Pan troglodytes	-17.0	-15.9	-9.4	-7.0	-11.0	-11.1	-18.3	-16.6	-10.6	-9.7	-16.2	-18.8	-15.8	-11.1	-17.9
**51 nt**															
Homo sapiens	-9.5	-7.3	-3.7	-2.8	-2.0	-4.4	-9.5	-10.0	-7.6	-7.6	-8.6	-9.6	-12.0	-7.9	-6.5
Denisova	-9.5	-7.3	-3.7	-2.8	-2.7	-4.4	-9.5	-10.3	-7.6	-7.6	-8.6	-9.6	-12.0	-7.9	-6.5
Pan troglodytes	-7.3	-8.1	-3.7	-2.9	-2.0	-4.3	-11.1	-11.6	-7.9	-5.1	-9.3	-9.6	-10.2	-6.7	-7.3
**25 nt**															
Homo sapiens	-1.1	-2.3	1.7	-0.6	-1.0	0.1	-1.3	0.3	-0.7	-1.2	-1.9	-9.6	-1.5	-4.9	-2.6
Denisova	-1.1	-2.3	1.7	-0.6	-1.0	0.1	-1.3	-3.4	-0.7	-1.2	-1.9	-9.6	-1.5	-4.9	-2.6
Pan troglodytes	1.1	-3.7	1.7	-1.5	-1.0	0.0	-1.7	-3.4	-0.9	-1.6	-1.9	-9.6	-0.9	-4.2	-2.7
ddG (Homo-Pan)															
301 nt	-1.6	-0.1	-0.6	-0.4	0.0	0.7	2.3	2.3	-0.2	-0.8	0.3	2.1	-5.7	-5.5	3.6
151 nt	0.2	-1.0	1.3	1.2	0.0	2.0	1.3	2.3	-2.7	-4.1	0.7	0.5	-5.1	-0.5	0.9
75 nt	-0.6	-0.7	-1.6	1.1	0.0	-0.1	1.6	1.6	-0.8	-2.9	0.7	1.8	-5.5	-0.6	-0.9
51 nt	-2.2	0.8	0.0	0.1	0.0	-0.1	1.6	1.6	0.3	-2.5	0.7	0.0	-1.8	-1.2	0.8
25 nt	-2.2	1.4	0.0	0.9	0.0	0.1	0.4	3.7	0.2	0.4	0.0	0.0	-0.6	-0.7	0.1
p-value	0.180	0.655	0.564	0.180		0.655	**0.025**	**0.025**	0.653	0.180	**0.046**	0.08	**0.025**	**0.025**	0.178
ddG (Homo-Den)															
301 nt	0	0	0	0	-0.4	0.3	0.3	0.3	0	0	0	0	0	0	0
151 nt	0	0	0	0	-0.8	2.5	1	1.2	0	0	0	0	0	0	0
75 nt	0	0	0	0	0	0	0.3	0.3	0	0	0	0	0	0	0
51 nt	0	0	0	0	0.7	0	0	0.3	0	0	0	0	0	0	0
25 nt	0	0	0	0	0	0	0	3.7	0	0	0	0	0	0	0
p-value					0.564	0.157	0.083	**0.025**							

Hs - Homo sapiens; Pt - Pan troglodytes.

Table S7. Gibbs free energy values for mRNA fragments of the *GRID2* gene in different species of primates, calculated by mFold [7].

Nucleotide position in CDS	18	129	138 & 141	228	457	528	711	879	1335	1593	1860	2217	2445
Branch	*Homininae*	*Homininae*	*Hominini*	*Hominoidea*	*Hominoidea*	*Homininae*	*Hominoidea*	*Hominini*	*Homininae*	*Hominoidea*	*Homininae*	*Hominoidea*	*Homininae*
**301 nt**							*dG (kcal/mol)*						
Homo sapiens		-84.50		-88.10	-76.50	-71.50	-66.70	-77.00	-82.20	-84.00	-79.20	-80.80	-80.30
Pan troglodytes		-82.90		-88.10	-76.10	-71.40	-70.50	-76.20	-84.40	-81.00	-81.30	-80.80	-80.30
Pan panicus		-82.90		-86.50	-76.10	-72.40	-70.50	-76.20	-85.80	-84.20	-81.30	-80.80	-80.30
Gorilla gorilla		-82.40		-86.20	-76.10	-72.40	-68.00	-76.90	na	na	-85.40	-80.80	-80.30
Pongo abelii		-83.00		-88.10	-81.20	-70.30	-68.70	-74.40	-87.40	-82.10	-78.40	-83.00	-75.20
Nomascus leucogenys		-80.30		-87.90	-76.60	-70.30	-67.70	-74.30	-90.10	-82.10	-83.10	-80.80	-80.30
Papio anubis		-82.40		-88.00	-78.70	-68.70	-68.30	-76.00	-86.30	-84.80	-84.90	-82.50	-79.40
Macaca fascicularis		-81.70		-88.00	-75.00	-68.40	-66.80	-76.30	-86.20	-83.50	-85.50	-79.60	-78.60
Colobus angolensis		-84.50		-88.00	-75.40	-69.80	-71.70	-76.20	-86.20	-83.20	-85.60	-82.20	-80.30
**151 nt**													
Homo sapiens	-35.80	-32.90		-39.00	-33.40	-30.80	-29.90	-36.50	-39.00	-33.50	-36.10	-38.50	-32.60
Pan troglodytes	-36.00	-32.90		-39.00	-33.40	-30.10	-29.90	-36.50	-39.00	-32.90	-36.10	-38.50	-32.60
Pan panicus	-36.00	-32.90		-39.00	-33.40	-30.10	-29.90	-36.50	-39.00	-32.90	-36.10	-38.50	-32.60
Gorilla gorilla	-34.30	-32.60		-39.00	-33.40	-30.10	-29.90	-38.00	na	na	-36.10	-38.50	-32.60
Pongo abelii	-35.20	-32.60		-39.00	-33.40	-29.40	-30.40	-38.00	-41.40	-32.90	-40.10	-38.50	-32.00
Nomascus leucogenys	-34.80	-30.40		-39.50	-33.40	-29.40	-28.70	-36.30	-44.00	-32.90	-39.10	-38.50	-32.00
Papio anubis	-37.00	-31.70		-40.30	-33.20	-33.30	-31.30	-37.60	-40.20	-37.20	-37.60	-35.20	-32.60
Macaca fascicularis	-35.80	-34.00		-40.30	-34.00	-33.60	-31.30	-38.00	-40.20	-36.30	-41.20	-30.70	-32.00
Colobus angolensis	-36.10	-34.00		-40.30	-33.20	-33.20	-31.30	-38.00	-40.20	-37.40	-38.80	-38.40	-35.50
**75 nt**													
Homo sapiens	-17.60	-11.90	-11.80	-12.90	-11.30	-14.90	-13.70	-16.40	-16.80	-10.80	-19.40	-14.90	-14.10
Pan troglodytes	-17.00	-11.90	-11.80	-12.90	-11.30	-14.90	-13.70	-16.40	-16.80	-11.50	-21.20	-14.90	-14.10
Pan panicus	-17.00	-11.90	-11.80	-12.90	-11.30	-14.90	-13.70	-16.40	-16.80	-11.50	-21.20	-14.90	-14.10
Gorilla gorilla	-17.60	-10.40	-11.10	-12.90	-11.30	-14.90	-13.70	-15.20	na	-11.50	-19.30	-14.90	-14.10
Pongo abelii	-14.90	-9.80	-13.00	-12.90	-10.90	-13.80	-13.70	-15.20	-19.70	-11.50	-24.10	-14.90	-14.10
Nomascus leucogenys	-16.70	-9.80	-13.00	-12.90	-10.90	-13.80	-13.70	-15.20	-22.30	-11.50	-24.50	-14.90	-14.10
Papio anubis	-16.90	-11.90	-13.00	-12.60	-9.90	-12.40	-16.00	-15.20	-18.50	-16.10	-24.80	-15.00	-14.10
Macaca fascicularis	-16.10	-9.80	-13.00	-12.60	-9.90	-12.40	-16.00	-15.20	-18.50	-16.10	-24.20	-15.00	-14.10
Colobus angolensis	-16.70	-9.80	-13.00	-12.60	-9.90	-12.40	-16.00	-15.20	-18.50	-16.10	-24.20	-15.00	-13.00
**51 nt**													
Homo sapiens	-6.60	-9.10	-9.20	-10.00	-7.00	-8.80	-6.00	-10.80	-11.20	-9.20	-13.10	-6.70	-10.50
Pan troglodytes	-5.70	-9.10	-9.20	-10.00	-7.00	-8.80	-6.00	-10.80	-11.20	-9.20	-13.10	-6.70	-10.50
Pan panicus	-5.70	-9.10	-9.20	-10.00	-7.00	-8.80	-6.00	-10.80	-11.20	-9.20	-13.10	-6.70	-10.50
Gorilla gorilla	-6.60	-7.60	-7.70	-10.00	-7.00	-8.80	-6.00	-10.70	na	-9.20	-13.10	-6.70	-10.50
Pongo abelii	-5.70	-7.00	-8.30	-10.00	-6.60	-9.50	-6.00	-10.70	-14.40	-9.20	-15.60	-6.70	-9.80
Nomascus leucogenys	-5.70	-7.00	-8.30	-10.00	-6.60	-9.50	-6.00	-10.70	-17.00	-9.20	-16.00	-6.70	-9.80
Papio anubis	-6.80	-7.00	-8.30	-9.70	-5.00	-7.90	-5.30	-10.70	-13.20	-9.50	-15.60	-6.50	-9.80
Macaca fascicularis	-6.80	-7.00	-8.30	-9.70	-4.80	-7.90	-5.30	-10.70	-13.20	-9.50	-15.70	-6.50	-9.80
Colobus angolensis	-6.80	-7.00	-8.30	-9.70	-5.00	-7.90	-5.30	-10.70	-13.20	-9.50	-15.70	-6.50	-9.80
**25 nt**													
Homo sapiens	-0.60	-0.30	-3.00	-2.40	0.20	-2.10	-0.30	-2.80	-3.30	-5.90	-6.70	-3.00	0.60
Pan troglodytes	-0.60	-0.30	-3.00	-2.40	0.20	-2.10	-0.30	-2.80	-3.30	-5.90	-6.70	-3.00	0.60
Pan panicus	-0.60	-0.30	-3.00	-2.40	0.20	-2.10	-0.30	-2.80	-3.30	-5.90	-6.70	-3.00	0.60
Gorilla gorilla	-0.60	-0.30	-2.50	-2.40	0.20	-2.10	-0.30	-0.60	-3.30	-5.90	-6.70	-3.00	0.60
Pongo abelii	-0.60	-3.50	-2.60	-2.40	-1.90	-0.30	-0.30	-0.60	-3.00	-5.90	-5.80	-3.00	0.60
Nomascus leucogenys	-0.60	-3.50	-2.60	-2.40	-1.90	-0.30	-0.30	-0.60	-3.00	-5.90	-5.90	-3.00	0.60
Papio anubis	-0.60	-3.50	-2.60	2.40	-1.90	-0.30	0.40	-0.60	-3.00	-4.10	-5.80	-3.10	0.60
Macaca fascicularis	-0.60	-3.50	-2.60	2.40	-1.90	-0.30	0.40	-0.60	-3.00	-4.10	-1.10	-3.10	0.60
Colobus angolensis	-0.60	-3.50	-2.60	2.40	-1.90	-0.30	0.40	-0.60	-3.00	-4.10	-1.10	-3.10	0.60

Table S8. Gibbs free energy values for mRNA fragments of the *GRID2* gene and their change depending on nucleotide variants in different primate taxa, calculated by mFold [7]. Species from taxon were taken with minimal number of specific variants which are not in other species from this taxon.

Nucleotide position in CDS	18	129	138 & 141	228	457	528	711	879	1335	1593	1860	2217	2445
Taxon with mutated allele	Homininae	Homininae	Hominini	Hominoidea	Hominoidea	Homininae	Hominoidea	Hominini	Homininae	Hominoidea	Homininae	Hominoidea	Homininae
Species from taxon	*Gorilla*	*Gorilla*	*H.sap*	*Pongo*	*Nomascus*	*Gorilla*	*Gorilla*	*H.sap*	*P.trogl*	*P.trogl*	*P.trogl*	*Nomascus*	*Gorilla*
dG (kcal/mol)													
301 nt	-82.40	-82.40	-84.50	-88.10	-76.60	-72.40	-68.00	-77.00	-84.40	-81.00	-81.30	-80.80	-80.30
151 nt	-34.30	-32.60	-32.90	-39.00	-33.40	-30.10	-29.90	-36.50	-39.00	-32.90	-36.10	-38.50	-32.60
75 nt	-17.60	-10.40	-11.80	-12.90	-10.90	-14.90	-13.70	-16.40	-16.80	-11.50	-21.20	-14.90	-14.10
51 nt	-6.60	-7.60	-9.20	-10.00	-6.60	-8.80	-6.00	-10.80	-11.20	-9.20	-13.10	-6.70	-10.50
25 nt	-0.60	-0.30	-3.00	-2.40	-1.90	-2.10	-0.30	-2.80	-3.30	-5.90	-6.70	-3.00	0.60
Taxon with ancestral allele	Hominoidea	Hominoidea	Homininae	Cercopithecidae	Cercopithecidae	Hominoidea	Cercopithecidae	Homininae	Hominoidea	Cercopithecidae	Hominoidea	Cercopithecidae	Hominoidea
Species from taxon	*Nomascus*	*Nomascus*	*Gorilla*	*Colobus*	*Colobus*	*Nomascus*	*Colobus*	*Gorilla*	*Pongo*	*Maccaca fas*	*Pongo*	*Colobus*	*Nomascus*
dG (kcal/mol)													
301 nt	-80.30	-80.30	-82.40	-88.00	-75.40	-70.30	-71.70	-76.90	-87.40	-83.50	-78.40	-82.20	-80.30
151 nt	-34.80	-30.40	-32.60	-40.30	-33.20	-29.40	-31.30	-38.00	-41.40	-36.30	-40.10	-38.40	-32.00
75 nt	-16.70	-9.80	-11.10	-9.70	-5.00	-13.80	-16.00	-15.20	-19.70	-16.10	-24.10	-6.50	-14.10
51 nt	-5.70	-7.00	-7.70	-9.70	-5.00	-9.50	-5.30	-10.70	-14.40	-9.50	-15.60	-6.50	-9.80
25 nt	-0.60	-3.50	-2.50	2.40	-1.90	-0.30	0.40	-0.60	-3.00	-4.10	-5.80	-3.10	0.60
ddG (kcal/mol)													
Species comparison	*Gg-Nl*	*Gg-Nl*	*Pt-Gg*	*Pa-Ca*	*Nl-Ca*	*Gg-Nl*	*Gg-Ca*	*Pt-Gg*	*Pt-Pa*	*Pt-Mf*	*Pt-Pa*	*Nl-Ca*	*Gg-Nl*
301 nt	-2.10	-2.10	-2.10	-0.10	-1.20	-2.10	3.70	0.70	3.00	1.10	-2.90	1.40	0.00
151 nt	0.50	-2.20	-0.30	1.30	-0.20	-0.70	-1.20	1.50	2.40	3.40	4.00	-0.10	-0.60
75 nt	-0.90	-0.60	-0.70	-0.30	-1.00	-1.10	2.30	-1.20	2.90	4.60	2.90	0.10	0.00
51 nt	-0.90	-0.60	-1.50	-0.30	-1.60	0.70	-0.70	-0.10	3.20	0.30	2.50	-0.20	-0.70
25 nt	0.00	3.20	-0.50	-4.80	0.00	-1.80	-0.70	-2.20	-0.30	-1.80	-0.90	0.10	0.00
p value	0.317	0.180	**0.025**	0.655	**0.046**	0.180	0.655	0.180	0.180	0.180	0.655	0.655	0.157

Gg - Gorilla gorilla; Nl - Nomascus leucogenys; Pt - Pan troglodytes; Pa - Pan panicus; Ca - Colobus angolensis; Mf - Macaca fascicularis.

Table S9. Comparison of the clinical characteristics of patients with different mutations in *GRID2* gene.

Article	Mutation in *GRID2*	Gait	Speech	Cognition and memory	Ocular motor signs and visual	Neurological signs and muscle tone	Other notes
Maier et al. 2013 [8]	Deletion of exon 1 (de novo)	Bipedal; walk limited to 10 meters	Dysarthria	Attention and anterograde memory deficits; information processing speed was significantly reduced	-	Spastic paraplegia; increased tendon reflex and palmomental reflex; Babinski signs; dysmetria; dysphagia	-
Van Schil et al. 2014 [9]	Deletion of exon 2 (homoz.)	-	Dysarthria	-	Oculomotor apraxia; difficulties in fixing gaze; nystagmus severe reduction in rod and cone responses	Abnormal backward head movements; truncal and appendicular ataxia; hypotonia; normal deep reflexes; slight ankle flexion and inversion contractures	Pectus excavatum deformity; some joint laxity
Veerapandiyan et al. 2017 [10]	Deletion of exon 2 (homoz.)	Bipedal; ability to walk without support at 7 y.o.	Speech delay and limited vocabulary	Development delay; the degree of cognitive impairment is not specified	Intermittent tonic upward gaze; vertical and horizontal nystagmus on eye movements; oculomotor apraxia (saccadic initiation failure and impaired voluntary pursuit)	Gross motor delays; severe central hypotonia; hyperreflexia; limb and truncal ataxia	Thoracolumbar kyphosis and scoliosis
Utine et al. 2013 [11]	Deletion of exons 3 and 4 (homoz.)	Bipedal; but unable to walk independently at 8 y.o. and 11 y.o.	Dysarthria	Global development delay; cognitive and memory impairment is not mentioned	Nystagmus; oculomotor apraxia; inability to fix the gaze; unable to name colors	Abnormal backward head movements; truncal ataxia; dysmetria; dysdiadochokinesia; increased tendon reflex and bilateral Babinski reflex	Pectus excavatum deformity; joint laxity; prominent calcaneus; subcutaneous fatty tissue increase bilaterally in the feet and prominent ears
Hill et al. 2013 [12]	Deletion of exon 4 (heteroz.) and exon 2 (homoz.)	Bipedal; but walk only with support; wheelchair-dependent	Single words and short sentences	Global development delay: speech and cognitive development	Nystagmus; a staring gaze; tonic upgaze	Truncal. appendicular ataxia; hypotonia	Joint laxity; no dysmorphic facial features
Coutelier et al. 2015 [13]	Missense mutation (c.1966C.G/p. Leu656Val) (heteroz/homoz)	Bipedal with instability signs (in 3 of 7 heteroz.); unable to walk independently at 7 and 5 y.o. (homoz.)	Limited; dysarthria (in 5 of heteroz. and homoz.)	Cognitive impairment (in 3 of 7 heteroz carriers and homoz); attention difficulties; visual memory deficit; low IQ	Abnormal saccadic pursuit (in 5 of 7 heteroz and homoz); nystagmus (in 2 of 7 heteroz)	Progressive cerebellar ataxia (slow progression in most carriers); hypotonia with brisk rotatinal reflex and pyramidal signs (in homoz)	Asymptomatic (2 of 7 heteroz). hearing loss (in 3 of 7 heteroz)
	2 missense mutations (c.1960G>A/p.Ala654Thr. c.1961C>A/p. Ala654Asp)	Bipedal; ability to walk only with support at 3 and 7 y.o.	Able to speak in complete sentences; dysarthria	No impairment	Abnormal saccadic pursuit; strabismus; nystagmus	Motor delay; progressive cerebellar ataxia; hypotonia	-
Ali et al. 2017 [14]	Missense mutation (c.2128C > T. p.Arg710Trp. homoz)	Bipedal; ability to walk without support at 7-8 y.o.	Dysarthria. limited vocabulary (≈100 words). often without logic	Development delay; Moderate intellectual disability	No signs; normal vision	Slowly progressive cerebellar ataxia; increased peripheral muscle tone; ataxic gait; dysmetria; dysdiadochokinesis	-
Ceylan et al. 2020 [15]	Duplication of exon 14 and possibly creating a premature stop codon	Ability to walk with support at 5 y.o.	Not mentioned	Mild intellectual disability	Ocular muscle weakness; nystagmus and oculomotor apraxia	Truncal ataxia; head titubation; dysmetria; dysdiadochokinesia and wide-based gait; increased peripheral muscle tone	Thoracic kyphoscoliosis, distal hyperlaxity

	(p.Ile788*). homoz.						
Iodice et al. 2020 [16]	Deletion of the exons.12 and 13; nonsense mutation (c.2564G > A, p.Ile788*). compound variants	Ability to walk with support at 18 m.o.	Slight language delay with dysarthria	Not available	Nystagmus; with age: ocular flutter. oculomotor apraxia and bilateral exophoria	Motor delay; ataxia; action tremor; truncal oscillations; hypotonia; mild distal hypotrophy in lower limbs without pyramidal signs.	-
Taghdiri et al. 2019 [17]	Deletion of the exons 3 to 16 (homoz.)	Ability to walk with support at 6 y.o.	Speech delay. Nasal and with stuttered speech	Developmental delay with the lowest amount of intellectual disability	Horizontal nystagmus	Truncal and appendicular ataxia; reduced force of extremities; slightly spastic muscle tone; increased deep tendon reflexes in lower extremities; signs of pyramidal involvement; impaired finger to nose test	Bilateral pes planus and hallux valgus
Hetzelt et al. 2020 [18]	Nonsense mutation (c.568C > T. p.Gln190* homoz.)	Not available	No speech, only syllables	Severe psychomotor development delay; very little interaction	Nystagmus; tonic upgaze; oculomotor apraxia; incomplete loss of sight	Severe and progressive truncal and appendicular ataxia; generalized muscular hypotonia; no head control; could not turn or sit, central hearing loss	Dystrophic; no facial dysmorphisms were noted
Brazilian case	Deletion of exons 5-7. (homoz.)	Quadrupedal	Speech is absent	Severe intellectual disability	Strabismus	Ataxia; seizures	Hirsutism; coarse facial features; short and small hands and feet; small stature; normal secondary sex characteristics

Homoz. – homozygous genotype; heteroz. – heterozygous genotype.

Supplemental Figures

Genome position	93225840	94031933	94031993	94137900	94145790	94159584	94159638	94316763	94344032	94344060	94376878	94411797	94436426	94436547	94690593
rs					rs373866971			rs1385405	rs72668716						
Nucleotide position in CDS	32	564	624	801	989	1188	1242	1251	1458	1488	1611	1866	2058	2178	2592
Amino acid substitution	S11F				E330M										
Population								G>T	T>C						
All					0.00004			0.244	0.023						
MSL								0.012	0.000						
ASW								0.082	0.000						
CHB								0.316	0.000						
GBR								0.357	0.022						
PEL								0.529	0.006						
STU								0.201	0.078						
Homo sapiens	C	C	T	T	C	C	A	T	C	C	G	A	G	G	A
Homo neanderthalensis	C	C	T	T	C	C	A	T	C	C	G	A	G	G	A
Denisova	C	C	T	T	Y	C	A	G	C	C	G	A	G	G	A
Pan troglodytes	T	G	C	C	C	T	C	G	Y	T	A	G	A	A	G
Pan panicus	T	G	C	C	C	T	C	G	C	T	A	G	G	A	G
Macaca Nemestrina	C	G	C	T	C	C	C	G	T	T	A	G	A	A	A
Colobus Angolensis	C	G	C	T	C	C	C	G	T	T	A	G	A	A	A
Aotus nancymae	T	G	C	T	C	C	C	G	T	T	A	G	A	A	A
Mus musculus	C	A	C	T	C	C	T	G	T	T	A	A	A	A	A
Ochotona princeps	C	G	C	T	C	C	C	G	T	C	C	G	A	A	T
Canis lupus	C	G	C	T	C	C	C	G	T	C	A	G	A	A	A
Dasypus novemcinctus	C	G	C	T	C	T	C	A	T	T	A	G	A	A	A
Echinops telfairi	C	A	C	T	C	T	A	G	T	T	A	A	A	A	A
Monodelphis domestica	T	G	C	T	C	A	T	C	T	T	A	A	A	A	A
Melopsittacus undulatus	C	A	C	T	C	G	C	C	C	T	A	G	A	T	A
Struchio camelus	C	G	C	T	C	T	C	C	T	T	A	A	A	C	A
Chrysemys picta	C	A	C	T	C	C	C	C	T	T	A	A	A	A	A
Anolis carolinensis	C	A	C	T	C	T	C	C	T	T	G	G	A	G	T
Xenopus tropicalis	C	C	C	T	C	A	C	C	T	T	A	G	A	A	A
Coelacanth	C	G	C	C	G	C	C	C	T	T	T	G	A	T	T
Larimichthys crocea	C	C	C	C	C	C	T	A	T	T	C	G	A	A	C
Esox lucius	C	G	C	T	C	A	C	C	T	T	G	A	A	G	C
Danio rerio	T	G	C	C	C	C	C	C	T	T	C	A	C	A	A

Figure S1. Evolutionary analysis of nucleotide substitutions in CDS of *GRID2* gene in *Homo sapiens* and close relatives. In red — variants predicted by mFold tools to change levels of Gibbs free energy in mRNA ($p<0.05$). In black — variants predicted by mFold tools as "not affecting" level of Gibbs free energy in mRNA (Table S6).

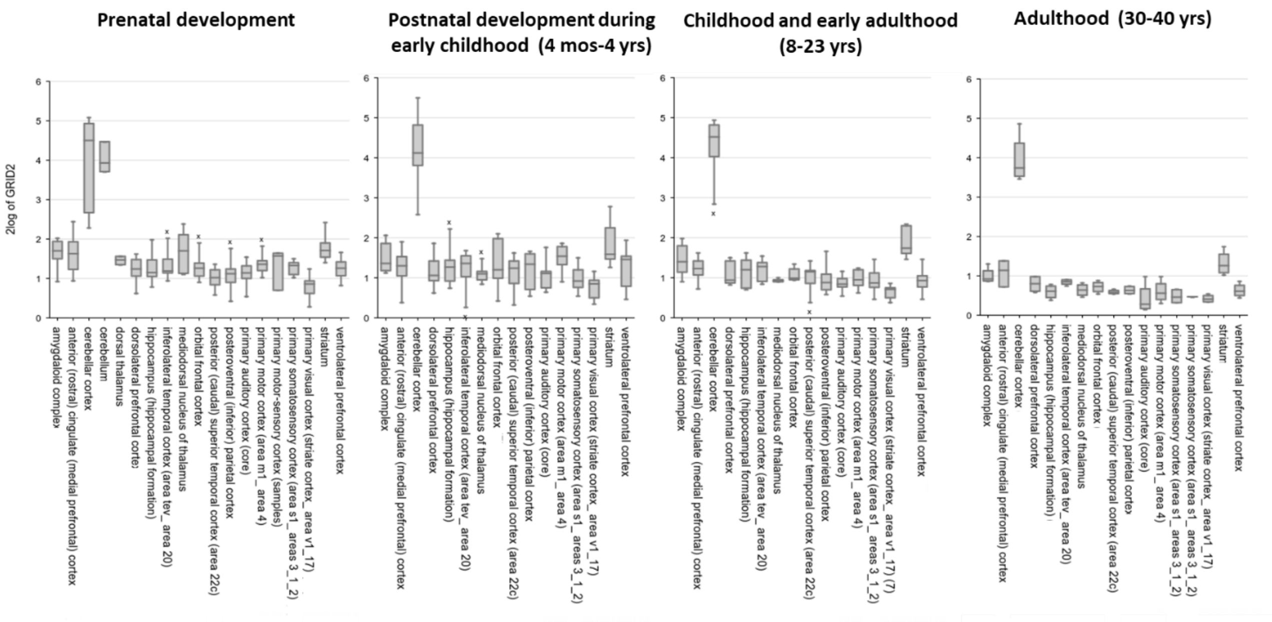

Figure S2. Expression of *GRID2* gene in different brain regions in *Homo sapiens* (BrainSpan [19]) visualized using R2: Genomics Analysis and Visualization Platform (http://r2.amc.nl).

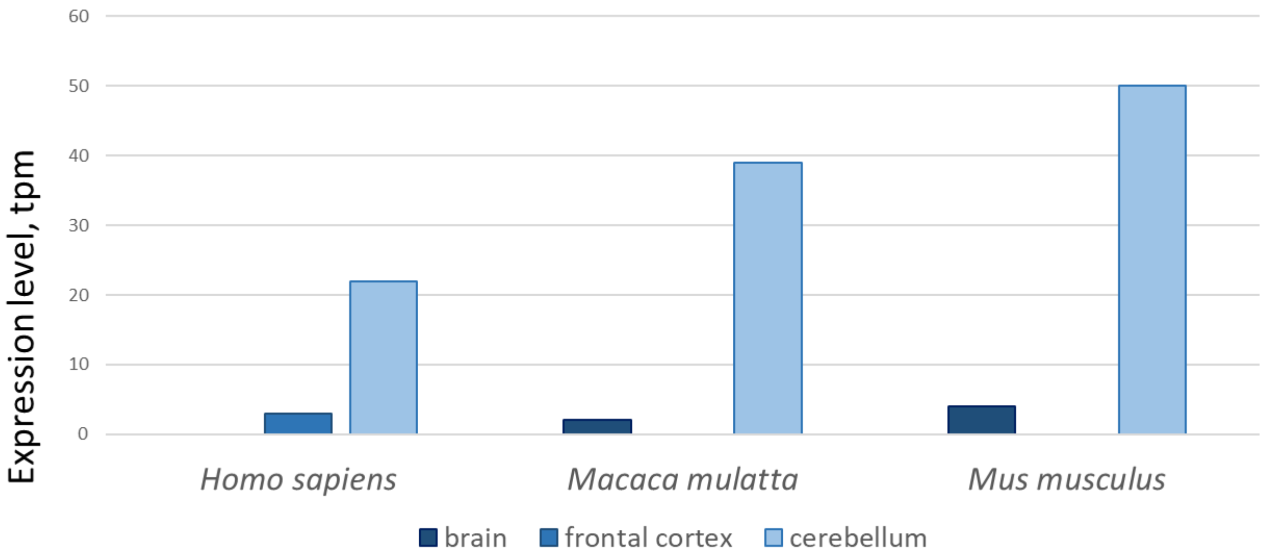

Figure S3. Expression of *GRID2* gene in the cerebellum in three mammalian species (obtained from Kaessmann lab mammalian gene expression data and Gene Expression atlas [20]).

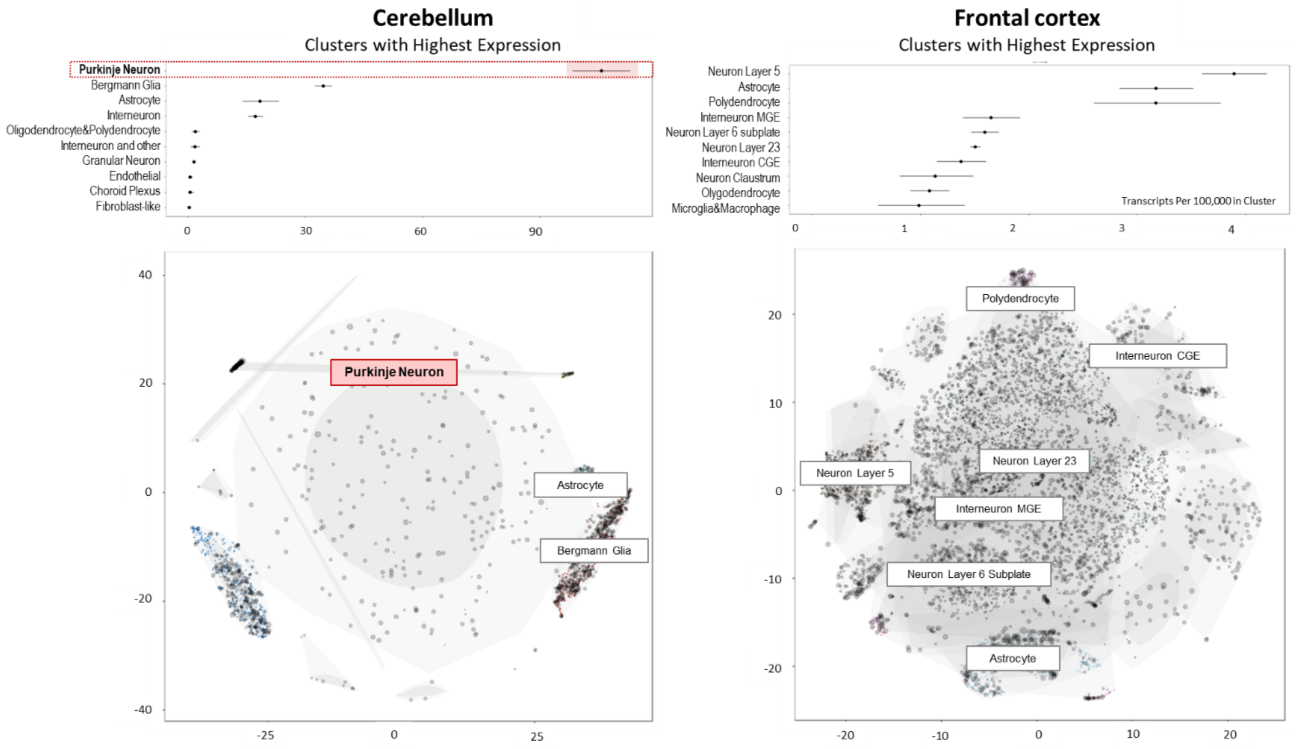

Figure S4. Single-cell expression of *GRID2* gene in the cerebellum and frontal cortex of adult mice (obtained from DropViz [21]).

Supplemental References

1. Seelow, D.; Schuelke, M.; Hildebrandt, F.; Nürnberg, P. HomozygosityMapper--an interactive approach to homozygosity mapping. *Nucleic. Acids. Res.* **2009**, *37*, W593–W599.
2. 1000 Genomes Project Consortium; Abecasis, G.R.; Auton, A.; Brooks, L.D.; DePristo, M.A.; Durbin, R.M.; Handsaker, R.E.; Kang, H.M.; Marth, G.T.; McVean, G.A. An integrated map of genetic variation from 1,092 human genomes. *Nature* **2012**, 491, 56–65.
3. Kircher, M.; Witten, D.M.; Jain, P.; O'Roak, B.J.; Cooper, G.M.; Shendure, J. A General Framework for Estimating the Relative Pathogenicity of Human Genetic Variants. *Nat. Genet.* **2014**, 46, 310–315, doi:10.1038/ng.2892.
4. Yang, Z. PAML: A program package for phylogenetic analysis by maximum likelihood. Comput. Appl. Biosci. 1997, 13, 555–556.
5. Yang, Z. PAML 4: Phylogenetic analysis by maximum likelihood. Mol. Biol. Evol. 2007, 24, 1586–1591.
6. Yang, Z.; Nielsen, R. Mutation-selection models of codon substitution and their use to estimate selective strengths on codon usage. *Mol. Biol. Evol.* **2008**, 25, 568–579.
7. Zuker, M. Mfold web server for nucleic acid folding and hybridization prediction. Nucleic Acids Res. 2003, 31, 3406–3415.
8. Maier, A.; Klopocki, E.; Horn, D.; Tzschach, A.; Holm, T.; Meyer, R.; Meyer, T. De novo partial deletion in GRID2 presenting with complicated spastic paraplegia. *Muscle Nerve* **2014**, 49, 289–292.
9. Van Schil, K.; Meire, F.; Karlstetter, M.; Bauwens, M.; Verdin, H.; Coppieters, F.; Scheiffert, E.; Van Nechel, C.; Langmann, T.; Deconinck, N.; et al. Early onset autosomal recessive cerebellar ataxia associated with retinal dystrophy: New human hotfoot phenotype caused by homozygous GRID2 deletion. *Genet. Med.* **2015**, *17*, 291–299.
10. Veerapandiyan, A.; Enner, S.; Thulasi, V.; Ming, X. A Rare Syndrome of GRID2 Deletion in 2 Siblings. *Child Neurol. Open* **2017**, *4*, 2329048X17726168.
11. Utine, G.E.; Haliloğlu, G.; Salanci, B.; Çetinkaya, A.; Kiper, P.Ö.; Alanay, Y.; Aktas, D.; Boduroğlu, K.; Alikaşifoğlu, M. A homozygous deletion in GRID2 causes a human phenotype with cerebellar ataxia and atrophy. *J. Child Neurol*. **2013**, *28*, 926–932.
12. Hills, L.B.; Masri, A.; Konno, K.; Kakegawa, W.; Lam, A.-T.N.; Lim-Melia, E.; Chandy, N.; Hill, R.S.; Partlow, J.N.; Al-Saffar, M.; et al. Deletions in GRID2 lead to a recessive syndrome of cerebellar ataxia and tonic upgaze in humans. *Neurology* **2013**, *81*, 1378–1386.
13. Coutelier, M.; Burglen, L.; Mundwiller, E.; Abada-Bendib, M.; Rodriguez, D.; Chantot-Bastaraud, S.; Rougeot, C.; Cournelle, M.-A.; Milh, M.; Toutain, A.; et al. GRID2 mutations span from congenital to mild adult-onset cerebellar ataxia. *Neurology* **2015**, *84*, 1751–1759.
14. Ali, Z.; Zulfiqar, S.; Klar, J.; Wikström, J.; Ullah, F.; Khan, A.; Abdullah, U.; Baig, S.; Dahl, N. Homozygous GRID2 missense mutation predicts a shift in the D-serine binding domain of GluD2 in a case with generalized brain atrophy and unusual clinical features. *BMC Med. Genet.* **2017**, *18*, 144.
15. Ceylan, A.C.; Acar Arslan, E.; Erdem, H.B.; Kavus, H.; Arslan, M.; Topaloğlu, H. Autosomal recessive spinocerebellar ataxia 18 caused by homozygous exon 14 duplication in GRID2 and review of the literature. *Acta Neurol. Belg*. **2020**, *121*, 1457–1462.
16. Iodice, A.; Spagnoli, C.; Cangini, M.; Soliani, L.; Rizzi, S.; Salerno, G.G.; Frattini, D.; Pisani, F.; Fusco, C. Long-term follow-up in infantile-onset SCAR18: A case report. *J. Clin. Neurosci.* **2020**, *77*, 232–234.
17. Taghdiri, M.; Kashef, A.; Abbassi, G.; Moshtagh, A.; Sadatian, N.; Fardaei, M.; Najafi, K.; Kariminejad, R. Further delineation of the phenotype caused by a novel large homozygous deletion of GRID2 gene in an adult patient. *Clin. Case Rep.* **2019**, *7*, 1149–1153.
18. Hetzelt, K.L.M.L.; Kraus, C.; Kusnik, S.; Thiel, C.T.; Uebe, S.; Ekici, A.B.; Trollmann, R.; Reis, A.; Zweier, C. A case of severe autosomal recessive spinocerebellar ataxia type 18 with a novel nonsense variant in GRID2. *Eur. J. Med. Genet.* **2020**, *63*, 103998.
19. Miller, J.A.; Ding, S.-L.; Sunkin, S.M.; Smith, K.A.; Ng, L.; Szafer, A.; Ebbert, A.; Riley, Z.L.; Royall, J.J.; Aiona, K.; et al. Transcriptional landscape of the prenatal human brain. *Nature* **2014**, *508*, 199–206.
20. Cardoso-Moreira, M.; Halbert, J.; Valloton, D.; Velten, B.; Chen, C.; Shao, Y.; Liechti, A.; Ascenção, K.; Rummel, C.; Ovchinnikova, S.; et al. Gene Expression across Mammalian Organ Development. Nature 2019, 571, 505–509, doi:10.1038/s41586-019-1338-5.
21. Saunders, A.; Macosko, E.Z.; Wysoker, A.; Goldman, M.; Krienen, F.M.; de Rivera, H.; Bien, E.; Baum, M.; Bortolin, L.; Wang, S.; et al. Molecular Diversity and Specializations among the Cells of the Adult Mouse Brain. Cell 2018, 174, 1015-1030.e16
